# The catechol-O-methyltransferase gene (*COMT*) and cognitive function from childhood through adolescence

**DOI:** 10.1016/j.biopsycho.2012.11.007

**Published:** 2013-02

**Authors:** Darya Gaysina, Man K. Xu, Jennifer H. Barnett, Tim J. Croudace, Andrew Wong, Marcus Richards, Peter B. Jones, the LHA genetics group

**Affiliations:** a MRC Unit for Lifelong Health and Ageing, University College London, London, UK; b Department of Psychiatry, University of Cambridge, Cambridge, UK; c School of Psychology, University of Leicester, Leicester, UK; d Cambridge Cognition Ltd, Cambridge, UK; e MRC/Wellcome Trust Behavioural and Clinical Neuroscience Institute, Cambridge, UK

**Keywords:** Dopamine, Birth cohort, Longitudinal study, Adolescent, Puberty

## Abstract

Genetic variation in the catechol-O-methyltransferase gene (*COMT*) can influence cognitive function, and this effect may depend on developmental stage. Using a large representative British birth cohort, we investigated the effect of *COMT* on cognitive function (verbal and non-verbal) at ages 8 and 15 years taking into account the possible modifying effect of pubertal stage. Five functional *COMT* polymorphisms, rs6269, rs4818, rs4680, rs737865 and rs165599 were analysed. Associations between *COMT* polymorphisms and cognition were tested using regression and latent variable structural equation modelling (SEM). Before correction for multiple testing, *COMT* rs737865 showed association with reading comprehension, verbal ability and global cognition at age 15 years in pubescent boys only. Although there was some evidence for age- and sex-specific effects of the *COMT* rs737865 none remained significant after correction for multiple testing. Further studies are necessary in order to make firmer conclusions.

## Highlights

► *COMT* gene can influence cognitive function in developmental stage specific manner. ► A large UK population-based sample with cognition measured at ages 8 and 15 years was used. ► Five functional *COMT* SNPs were tested for association with cognition. ► *COMT* rs737865 showed association with reading comprehension, verbal ability and global cognition at age 15 years in pubescent boys only. ► Further studies are necessary in order to make stronger conclusions.

## 1 Introduction

Genetic variation in the catechol-O-methyltransferase (*COMT*) gene is likely to be particularly important for phenotypes associated with function of the prefrontal cortex (PFC), such as cognition (Dickinson and Elvevag, 2009). Neuroimaging studies confirm that the *COMT* Val^158^Met (rs4680) polymorphism affects human prefrontal cortical function, and as such is strongly associated with differences in neural process underlying cognitive output (Dennis et al., 2010; Mier et al., 2009). However, these findings do not necessarily imply any change in cognition. A recent meta-analysis observed no significant effect of the Val^158^Met single nucleotide polymorphism (SNP) on frontal cognitive tasks (Barnett et al., 2008). Nevertheless, some studies have indicated that this association might be specific to developmental stage (Barnett et al., 2007; Dumontheil et al., 2011; Raz et al., 2011). Thus further examination of *COMT* genetic variation is required for a better understanding of its role in a wider range of cognitive functions during development.

Relatively little is known about the role of *COMT* in cognition in children (Diamond et al., 2004; Dumontheil et al., 2011), and specifically in relation to developmental stages, such as puberty (Barnett et al., 2007). The cognitive effects attributable to *COMT* activity may depend on developmental stage because structural and functional changes occur in the PFC during adolescence (Casey et al., 2000; De Luca et al., 2003; Rubia et al., 2000; Supekar et al., 2010); cognitive functions performed by the PFC in adults may be governed by different or more diffuse circuits in children. If so, then *COMT* variation may have little effect on cognitive performance during childhood, but a stronger effect in adolescence. Moreover, increases in the level of reproductive hormones such as estrogen during puberty can down-regulate *COMT* transcription and lead to a sex difference in *COMT* activity, and therefore to a different effect of *COMT* on cognition in boys and girls (Tunbridge, 2010). Among 8-year-old children from the Avon Longitudinal Study of Parents and Children (ALSPAC) cohort Val^158^Met polymorphism was reported to have a larger effect on verbal IQ in pubertal children when compared with prepubertal children (Barnett et al., 2007). A recent study of 6–20-year-olds also suggests the role of development in the effect of *COMT* Val^158^Met polymorphism on working memory, specifically that visuospatial working memory capacity exhibited an age by genotype interaction, with a benefit of the Met allele (rs4680) emerging after 10 years of age (Dumontheil et al., 2011). However, these studies were cross-sectional in design, so did not investigate the genetic effect on cognition in the same children at different developmental stages.

Despite strong evidence for the biological importance of several *COMT* SNPs (Nackley et al., 2006) little is known about associations with cognition of any loci other than Val^158^Met. A functional three-SNP haplotype consists of Val158Met (rs4680) and two synonymous SNPs (rs6269 and rs4818): ValA/ValA, ValA/Met, ValA/ValB or Met/Met, ValB/Met, and ValB/ValB are diplotypes ranked from highest to lowest according to *COMT* enzyme activity. This haplotype exerts a major influence on the level of *COMT* expression and enzyme activity (Nackley et al., 2006) and has previously been shown to have a curvilinear association with measures of verbal inhibition and working memory (Barnett et al., 2009). Previous studies have also reported associations between rs165599 (located near the 3′UTR region) (Burdick et al., 2007; Chan et al., 2005), and rs737865 (located in intron 1) (Diaz-Asper et al., 2008; Liao et al., 2009) and cognitive function. These SNPs appear to be functional, with rs737865 G (=C) allele and rs165599 G (=C) allele being associated with lower expression of *COMT* mRNA in the human brain (Bray et al., 2003).

In the present study we include the Nackley's haplotype (rs6269–rs4818–rs4680) and the two functional SNPs, rs737865 and rs165599, to characterize better the combined effects of variation in the *COMT* gene on cognitive function. Using data from the British 1946 birth cohort we aimed: (1) to investigate the effect of the five *COMT* SNPs on cognitive function in the same boys and girls at two time-points (age 8 and 15 year follow-ups); (2) to test whether pubertal stage of cohort members established at the second assessment (age 15 years) modifies any observed associations.

## 2 Methods

### 2.1 Sample

The Medical Research Council (MRC) National Survey of Health and Development (NSHD) (also known as the British 1946 birth cohort) is a socially stratified birth cohort of 5362 individuals (2547 women and 2815 men), who have been followed up since their birth in 1946 with regular data collections (Wadsworth et al., 2003). Blood samples were collected from 2756 members at age 53 years. Every survey member with information on at least one cognitive test (phenotype indicator) at both age 8 and age 15 years and DNA genotyped for *COMT* SNPs was included in the descriptive analysis (*n* = 1029 boys and 1048 girls). Survey members with available DNA had higher cognitive scores on all cognitive tests at ages 8 and 15 years than those without genetic information; but were not different with respect to pubertal stage at age 15 years (*p* = 0.35) or social class of origin (*p* = 0.52). The results of comparing those with DNA and those without on cognitive tests measures are presented in Supplementary Table 1.

Ethical approval for this research was obtained from the North Thames Multi-Centre Research Ethics Committee, and from relevant local research ethics committees in the survey areas. Informed consent was given by all respondents.

### 2.2 Measures

#### 2.2.1 Cognitive function

Children were assessed by teachers in a school setting at ages 8 and 15 years using tests devised by the National Foundation for Educational Research (Pigeon and Douglas, 1964; Pigeon et al., 1968). At age 8 years these were: (1) reading comprehension (selecting appropriate words to complete 35 sentences); (2) word reading (ability to read and pronounce 50 words); (3) vocabulary (ability to explain the meaning of the same 50 words); and (4) picture intelligence, consisting of a 60-item non-verbal reasoning test. At age 15 years these were: (1) Alice Heim Group Ability Test (AH4), a 130 item timed test, with separate verbal (analogies, comprehension, and numerical reasoning) and non-verbal (matching, spatial analysis, and non-verbal reasoning sections) tests; (2) The Watts-Vernon Reading Test, a test of reading comprehension requiring the participant to select appropriate words to complete 35 sentences; (3) A 47-item mathematics test, requiring the use of arithmetic, geometry, trigonometry, and algebra.

All scores reported in the results were standardised within the sample included in this analysis to a mean of 0 and a standard deviation of 1.

#### 2.2.2 Pubertal status

At age 15 years, pubertal development in boys was classified at school by a physician based on the development of genitalia, presence of pubic hair, axillary hair and voice broken. Those with infantile genitalia or early adolescent genitalia, but no pubic or axillary hair and voice not broken were classified as prepubescent, all others as pubescent. Age at menarche was used as the marker of pubertal stage for girls, and was obtained from mothers’ reports in 1961. This information was used to construct a binary variable for pubertal status distinguishing between those who had menarche by age 15 (pubescent) and those who did not (prepubescent). The majority of the participants (89% of boys and 90% of girls) had reached pubertal status by age 15.

### 2.3 Genotyping

DNA was extracted and purified from whole blood using the Puregene DNA Isolation Kit (Flowgen, Leicestershire, UK) according to the manufacturer's protocol. The five SNPs, rs737865, rs6269, rs4818, rs4680 and rs165599, were typed by using the KASPar system by KBioscience, UK (www.kbioscience.co.uk). The integrity of the genotyping was checked by genotyping frequency, concordance of duplicates and Hardy–Weinberg equilibrium (HWE). The call rates for the genotyped SNPs were 97.8–99.2%, with >95% concordance between duplicate samples and there was no evidence of deviation from HWE (*p* > 0.05).

The programme PLINK v1.07 was used for haplotype analysis (Purcell et al., 2007). The haplotype frequencies (rs6269–rs4818–rs4680) were similar to those reported in the original paper (Nackley et al., 2006): GGG (=ValA)–40.8%, ACA (=Met)–50.8%, ACG (=ValB)–7.8%, all others–0.6%. The survey members were then assigned to one of six possible diplotypes (i.e., the pair of haplotypes) using a ‘phase’ option (Table 1): ValA/ValA, ValA/Met, ValA/ValB or Met/Met, ValB/Met, and ValB/ValB.

### 2.4 Statistical analysis

Of the five genotyped *COMT* SNPs, the central three, rs6269, rs4818 and rs4680, were in high linkage disequilibrium (LD): *r*^2^ = 0.72 for rs6269–rs4680 and rs4818–rs4680; and *r*^2^ = 0.97 for rs6269–rs4818. In contrast, the LD between the three SNPs in the haplotype block and the other two SNPs was low (all *r*^2^ < 0.38). We therefore chose to separately test associations between cognitive scores and rs737865 and rs165599 and with the three-SNP haplotype.

First, linear regression was used to test for associations between rs737865, rs165599 genotypes (under an additive model), the three-SNP haplotype and the cognitive measures. In addition, curvilinear regression was used to test for associations between the three-SNP haplotype and the cognitive measures. Analyses were performed separately for boys and girls, and the sex-by-genotype interaction term was fitted to test for sex differences. At age 15 years, analyses were also stratified by pubertal stage, and the puberty-by-genotype interaction term was fitted to test differences between pubertal and pre-pubertal groups.

The effect of *COMT* genotypes on global cognitive function in a longitudinal context was examined using structural equation modelling (SEM) (Schumacker and Lomax, 2004). Model estimation was performed with Mplus version 6 (Muthen and Muthen, 2007). The model fit was evaluated with recommended fit indices (Hu and Bentler, 1999): the Tucker–Lewis index (TLI), the root mean square error of approximation (RMSEA), and the comparative fit index (CFI).

A graphical depiction of the model is shown in Fig. 2. The measurement part of the SEM model represents overall cognitive function at ages 8 and 15 years. The structural part includes direct paths from the *COMT* genotype to cognition at age 8 and age 15. We fitted the model using multiple group analysis (Byrne, 2004). In the final analytic model, the factor loadings of global cognition factors were constrained to be invariant across gender groups, whereas the path coefficients from *COMT* genotype to cognition were freely estimated.

The item intercepts were freely estimated in both groups because the main parameters of interest are path coefficients, hence invariance of factor loadings are sufficient (see (Gregorich, 2006), for a detailed discussion on level of measurement invariance). We performed the Wald *χ*^2^ test of parameter equalities for gender group differences in the structural regression paths.

Since SNPs associations were examined in multiple group SEM with cognition modelled as a latent variable, it was important to examine whether the measures of cognition were comparable across the gender groups (Byrne, 2004; Meredith, 1993). Two confirmatory factor analysis (CFA) models with different degrees of measurement parameter restrictions were specified in order to assess the extent to which the validity of the comparison of path coefficients across groups held. The baseline model tested configural invariance where latent global cognition variable had the same number of factor indicators, i.e. same number of items representing specific test domains in male and female groups. In this model, the factor loadings across gender groups were freely estimated. This model was a prerequisite for testing the next step, the metric invariance, where the factor loadings were constrained to be equal across groups. The measurement invariance in factor loadings ensures that the global cognition construct has the same substantive meaning across gender groups, thus warranting valid comparison of regression path coefficients in the SEM model. Then model fit indices of the two models were compared to evaluate the degree of measurement invariance of the loading parameters in the models.

We used the same range of the above-mentioned fit indices to investigate models of measurement invariance. The restrictive model is preferred if the fit indices are not significantly inferior compared to that of the less restrictive model. In terms of the RMSEA, the change should be less than .015 (Chen, 2007). For CFI, the change should be less than .01 in CFI (Chen, 2007; Cheung and Rensvold, 2001). We also presented the TLI and Chi-square as overall tests for goodness of fit (Marsh et al., 1998).

A Bonferroni correction was applied in an attempt to address the issue of multiple testing. The total number of independent tests was 15 (two individual SNPs plus one haplotype in two gender groups at two ages plus three tests [two SNPs and one haplotype] for pubertal status at age 15 in boys only). We did not treat each cognitive test as independent as they were highly inter-correlated (*r* = 0.6–0.9). This approach to inferences on independent tests required that the conservative α-level of 0.0033 to be used as the significance level for robust inferences.

## 3 Results

**Fig. 1:**
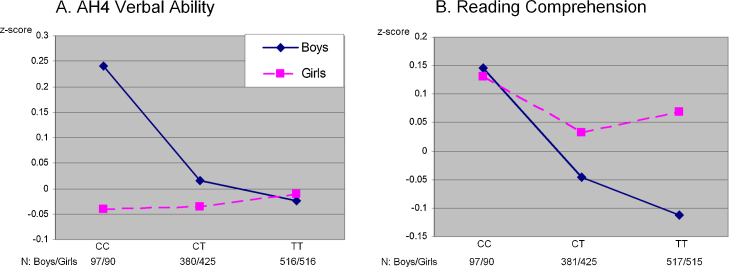
Sex-specific associations between *COMT* rs737865 and cognitive function at age 15 years.

Descriptive statistics for the phenotype measures and genetic data, by sex, are presented in Table 1. The results of association analysis between the *COMT* SNPs and cognitive traits at age 8 and 15 years are presented separately for boys and girls in Table 2. There were no associations between rs737865, or rs165599 or diplotype and cognitive measures at age 8 years in either boys or girls. The regression analysis for cognition at age 15 years identified associations between rs737865 and AH4 verbal ability (*β* = −0.106, SE = 0.049, *p* = 0.031), and reading comprehension (*β* = −0.098, SE = 0.049, *p* = 0.044), but only in boys (Fig. 1). Boys with CC genotype had higher scores than those–carriers of T allele (both *p*'s < 0.05). None of the findings survived *p*-value correction for multiple testing (α-level = 0.0033).

There were no associations between this SNP and level of cognitive functions in girls. Sex differences were tested by interaction terms but found not to be statistically significant at α-level of 0.0033: *p* for sex interaction = 0.13 and 0.09 for verbal ability and reading comprehension, respectively. There were no associations between diplotypes and any of the individual cognitive tests at age 15 years using linear (Table 2) or curvilinear (Supplementary Table 2) regression models.

The effect of pubertal stage on the associations between rs737865 and reading comprehension and verbal ability was then tested. In boys, we observed the similar effects of the SNP on verbal ability (*β* = −0.123, SE = 0.051, *p* = 0.016) and reading comprehension (*β* = −0.110, SE = 0.051, *p* = 0.031) in those who reached puberty by age 15 years. There were no associations between rs737865 and either verbal ability (*β* = 0.062, SE = 0.124, *p* = 0.62) or reading comprehension (*β* = −0.026, SE = 0.127, *p* = 0.84) in prepubescent boys. There was no statistically significant difference between prepubescent and pubescent groups: *p*-values for the puberty interaction tests were 0.18 and 0.17 for verbal ability and reading comprehension respectively.

**Fig. 2:**
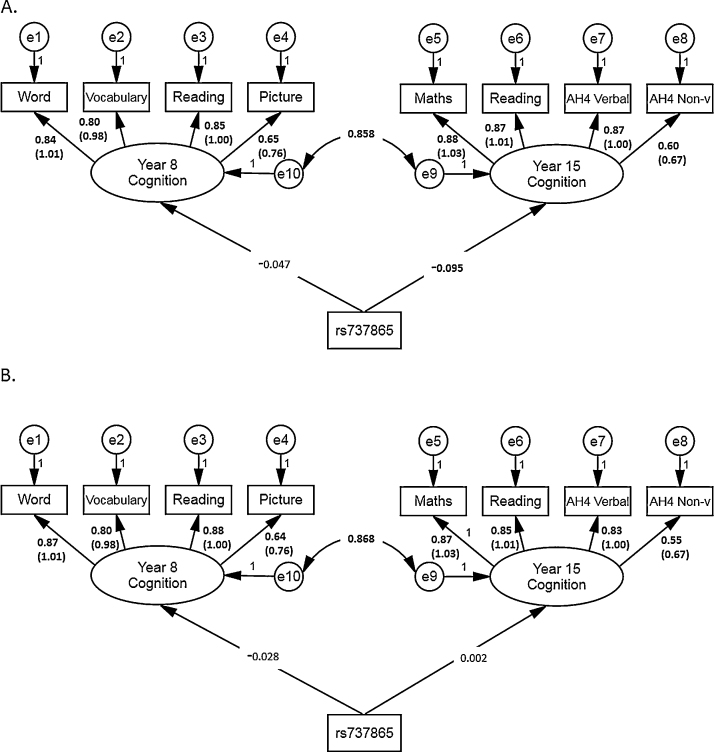
SEM of longitudinal effect of *COMT* rs737865 on cognition at ages 8 and 15 years in boys (*n* = 1134, (a) and girls (*n* = 1157, (b); word = word reading, reading = reading comprehension, picture = picture intelligence, AH4 verbal = AH4 verbal ability, AH4 non-v = AH4 non-verbal ability; coefficients with *p* < 0.05 are in bold. For factor loadings, both standardised and unstandardised (in brackets) coefficients are presented.

We tested for the longitudinal effect of *COMT* rs737865, rs165599 and diplotype on cognition using SEM model for boys and girls (Fig. 2 for rs737865). Individual cognitive tests were modelled as components of global cognition at both ages. Results of modelling to test for measurement invariance of global cognitive measures showed that the less restrictive model demonstrated a good fit to the data (chi2 = 512.944, df = 34, CFI = 0.980, TLI = 0.968, RMSEA = 0.080). The second model with more restrictive equal factor loadings across gender groups showed even better fit indices due to model parsimony (chi2 = 560.089, df = 40, CFI = 0.979, TLI = 0.970, RMSEA = 0.076), supporting the invariance of factor loadings across gender groups. This provided a sufficient condition for comparison of the *COMT* SNP association with global cognitive measures across both genders. A multiple group SEM model was fitted with rs737865 as predictor of the global cognition (TLI = 0.971, CFI = 0.979, RMSEA = 0.065). The results of the SEM estimation suggested the effect of rs737865 on general cognition at age 15 in boys only (Table 3; Fig. 2), although the results of the Wald test for gender group differences in the structural regression paths were non-significant (*χ*^2^ = 2.19, *p* = 0.14).

Stratified analysis showed that the association between rs737865 and global cognition at age 15 was significant in pubescent (*β* = −0.11, SE = 0.05, *p* = 0.037), but not in prepubescent (*β* = −0.01, SE = 0.16, *p* = 0.96) boys, although the Wald test revealed that the differences by developmental stage were not statistically significant (*χ*^2^ = 0.34, *p* = 0.56).

The results of the SEMs for rs165599 and diplotype provided no evidence for the association with cognitive function at age 8 or 15 years (Table 3).

## 4 Discussion

After correcting for multiple testing, the present study failed to demonstrate a significant effect of the five *COMT* SNPs on cognition in boys and girls at ages 8 and 15 years, providing little evidence that *COMT* variation can have an effect on cognitive abilities in childhood and adolescence.

However, several limitations should be taken into account when interpreting the present findings. Losses to follow-up and missing data are unavoidable in long running birth cohort studies such as the NSHD. There were differences in cognitive measures between those with DNA and those without DNA. This potentially could lead to underestimation of the effect of the *COMT* gene on cognition. However, that would be the case if the association operates differently in those with lower scores of cognitive abilities. We did not formally test for population stratification; however the 1946 birth cohort represents the general population of Britain of the middle of 20th century, which is of white Caucasian origin.

The strength of the study is its representative large sample. Yet, given the small number of prepubescent adolescents, there might still not be enough power to detect the small effect of the *COMT* individual SNPs or diplotypes in the groups stratified by pubertal status at age 15 years. In light of the possibility of the Type II error, the present results should be interpreted with caution. Indeed, the effect sizes of the original regressions were small (<1% of the common variance), and the results did not withstand correction for multiple testing.

Another strength of the study is the longitudinal analysis of the *COMT* gene in cognitive function using the SEM approach. The SEM approach allows for greater precision in phenotype measurement due to correction for measurement error in the cognitive outcomes, which may otherwise have reduced the statistical power to detect any robust associations with these genes (van der Sluis et al., 2012)

Before applying Bonferroni correction for multiple testing, only one *COMT* SNP, rs737865, was associated with verbal cognition in pubescent boys at age 15. Therefore, our finding did not confirm the results in the ALSPAC cohort showing a significant effect of *COMT* rs4680 on verbal IQ in pubescent boys (Barnett et al., 2007). The results of the analysis of *COMT* diplotypes on cognitive functions in children were not statistically significant and did not confirm the previous findings of curvilinear association between *COMT* diplotypes and cognition in ALSPAC cohort (Barnett et al., 2008).

We believe that testing for the effect of the *COMT* gene on various cognitive abilities is important for several reasons. It has been demonstrated that *COMT* protein has the strongest effect on the dopamine neurotransmission in the PFC, the brain region that plays an important role in a wide variety of cognitive functions, including cognitive control and IQ (Green et al., 2012). Moreover, performances on diverse tests of cognitive function tend to correlate; this underlying covariance represents general cognitive ability (‘g’). It is therefore logical that *COMT* could affect general intelligence as well as specific executive tasks (Duncan et al., 2000)

In our study, we were able to test the effect of the *COMT* gene on cognitive abilities at different developmental stages. It has been reported, that the heritability of general cognitive ability increases significantly and linearly from childhood through young adulthood (Haworth et al., 2010). In line with this observation, a recent study of 6–20-year-olds showed that visuospatial working memory capacity exhibited an age by genotype interaction with a benefit of the Met allele emerges during adolescence (Dumontheil et al., 2011).

The level of a reproductive hormone oestrogen, which down-regulates *COMT* transcription (Xie et al., 1999), increases during puberty in girls. This suggests that adolescence is an important developmental period, when the sex difference in *COMT* activity emerges (Xie et al., 1999), and the effect of *COMT* on cognitive abilities between boys and girls differentiates. Therefore, adolescence can be an important period when the sex difference in *COMT* activity emerges, and the effect of *COMT* on cognition between boys and girls differentiates. However, our study was not able to confirm the effect of *COMT* variation in adolescent girls or boys at age 15. It remains unclear whether the previously reported age-specific effect may be due to puberty. The puberty-by-gene interaction effects were not significant in boys, and there is a lack of power in our study since the group of prepubescent boys is small. On the other hand, many developmental changes may occur between ages 8 and 15 years, and young people are exposed to influences from many environmental factors. Therefore, we cannot exclude possible genotype-environment interaction on cognition. Recently, epigenetic mechanism for the interaction of the *COMT* with lifetime stress has been discovered: the greater stress led to lower methylation of the Val^158^ that was related to reduced cortical efficiency during a cognitive task (Ursini et al., 2011). Future studies will need to address these issues by employing longitudinal designs, using repeated measures of cognitive functions, and exploring the effects of specific DNA variants in interaction with environmental factors on trajectory of changes across cognitive development.

In conclusion, the present longitudinal study provides some evidence that *COMT* variation may affect cognitive function in a sex or developmental stage-specific manner. Further studies are necessary in order to make stronger conclusions.

## Figures and Tables

**Table 1:** Descriptives for cognitive phenotypes and *COMT* genotypes/diplotypes by sex.

Phenotype data	Boys		Girls		*p*
	*n* total	Mean (SD)	n total	Mean (SD)	
Cognition at age 8					
Word reading	1024	−0.07 (1.04)	1046	0.07 (0.96)	0.002
Vocabulary	1024	0.04 (1.00)	1046	−0.04 (1.00)	0.06
Reading comprehension	1024	−0.07 (1.03)	1046	0.07 (0.96)	0.001
Picture intelligence	1029	0.01 (1.01)	1046	−0.01 (1.00)	0.75
Cognition at age 15					
AH4 Verbal Ability	1027	0.02 (1.01)	1047	−0.02 (0.98)	0.26
Reading Comprehension	1029	−0.06 (1.02)	1047	0.06 (0.97)	0.007
Mathematics	1028	0.18 (1.06)	1047	−0.17 (0.91)	<0.0001
AH4 Non-verbal Ability	1028	0.09 (1.00)	1048	−0.09 (1.00)	<0.0001

Genotype data	*n*	Freq (%)	*n*	Freq (%)	*p*
*COMT* genotypes					
rs737865: CC/CT/TT	995	9.8/38.3/51.9	1031	8.7/41.2/50.1	0.36
rs6269: GG/AG/AA	1016	17.6/48.2/34.2	1038	17.0/46.9/36.1	0.64
rs4818: GG/CG/CC	1020	17.8/47.8/34.4	1037	16.6/47.2/36.2	0.62
rs4680: GG/AG/AA	1020	24.5/50.8/24.7	1034	23.1/50.4/26.5	0.58
rs165599: GG/AG/AA	1008	9.8/40.7/49.5	1028	9.2/41.3/49.4	0.89
*COMT* diplotypes*					
ValA/ValA	181	17.8	175	16.9	
ValA/Met	428	42	426	41.1	
ValA/ValB or Met/Met	315	31	334	32.3	
ValB/Met	86	8.5	93	9	
ValB/ValB	7	0.7	7	0.7	0.94

*Note*: *COMT* diplotype = rs6269–rs4818–rs4680; reference groups are CC–for rs737865, GG–for 165599, ValA/ValA–for Nackley's diplotype.

**Table 2:** Results of linear regression analysis for the association between *COMT* and cognitive function at ages 8 years and 15 years in boys and girls.

Cognitive measures	Boys									Girls								
	rs737865			rs165599			Diplotypes*			rs737865			rs165599			Diplotypes		
	*n*	β	*p*	*n*	β	*p*	*n*	β	*p*	*n*	β	*p*	*n*	β	*p*	*n*	β	*p*
Age 8																		
Word reading	990	−0.050	0.32	1003	−0.033	0.50	1012	−0.023	0.54	1029	−0.068	0.14	1026	−0.044	0.33	1033	−0.012	0.71
Vocabulary	990	−0.026	0.60	1003	−0.005	0.91	1012	−0.019	0.59	1029	−0.024	0.61	1026	−0.013	0.78	1033	−0.016	0.65
Reading comprehension	990	−0.083	0.09	1003	−0.009	0.86	1012	−0.017	0.64	1029	−0.014	0.76	1026	−0.013	0.78	1033	−0.002	0.96
Picture intelligence	995	0.006	0.89	1008	−0.003	0.96	1017	0.015	0.68	1029	−0.037	0.43	1026	−0.047	0.33	1033	−0.007	0.83

Age 15																		
Reading Comprehension	993	−0.098	0.044	1006	0.019	0.70	1015	−0.033	0.36	1031	0.019	0.70	1027	−0.013	0.78	1034	−0.030	0.39
AH4 Verbal Ability	995	−0.106	0.031	1008	−0.012	0.81	1017	−0.044	0.22	1030	−0.004	0.94	1027	−0.018	0.7	1034	0.011	0.75
AH4 Non-verbal Ability	994	−0.024	0.61	1007	0.042	0.38	1016	−0.007	0.85	1031	−0.023	0.63	1028	−0.067	0.16	1035	−0.039	0.26
Mathematics	994	−0.086	0.09	1007	−0.033	0.52	1016	−0.018	0.64	1030	−0.017	0.68	1027	−0.083	0.06	1034	−0.025	0.43

*Note*: *rs6269–rs4818–rs4680; reference groups are CC–for rs737865, GG–for 165599, ValA/ValA–for Nackley's diplotype. β based on standardized outcomes and unstandardized predictor.

**Table 3:** Results of SEM of longitudinal effect of *COMT* on global cognition at ages 8 and 15 years.

	Boys					Girls				
	*n*	Age 8		Age 15		*n*	Age 8		Age 15	
		β	*p*	β	*p*		β	*p*	β	*p*
rs737865	1134	−0.047	0.341	−0.095	0.046	1157	−0.028	0.568	0.002	0.971
rs165599	1146	0.018	0.718	0.012	0.802	1156	−0.037	0.449	−0.06	0.22
Diplotype*	1156	−0.01	0.792	−0.033	0.362	1163	−0.016	0.662	−0.029	0.415

*Note*: Diplotype = rs6269–rs4818–rs4680; reference groups are CC–for rs737865, GG–for 165599, ValA/ValA–for Nackley's diplotype. β based on standardised outcomes and unstandardized predictor.
