# Supplementary material for: The catechol-O-methyltransferase gene (COMT) and cognitive function from childhood through adolescence
Source: Biol Psychol. 2013 Feb;92(2):359–64. doi: 10.1016/j.biopsycho.2012.11.007 (PMC3580283; doi:10.1016/j.biopsycho.2012.11.007)
Supplement: Supplementary file 1 [file mmc1.docx]

Supplementary material

Supplementary Table 1. The analysis of differences in cognitive scores between those with DNA available and those without DNA

| **Cognitive measures** | DNA is available N=2077 | DNA is not available N=1766 | *P* |
| --- | --- | --- | --- |
| **Age 8** |  |  |  |
| Word reading | 0.049 (0.993) | -0.057 (1.006) | 0.001 |
| Vocabulary | 0.045 (0.996 | -0.053 (1.002) | 0.002 |
| Reading comprehension | 0.061 (0.981) | -0.072 (1.017) | <0.001 |
| Picture intelligence | 0.079 (0.964) | -0.093 (1.033) | <0.001 |
| **Age 15** |  |  |  |
| Reading Comprehension | 0.081 (0.939) | -0.096 (1.059) | <0.001 |
| AH4 Verbal Ability | 0.076 (0.953) | -0.089 (1.046) | <0.001 |
| AH4 Non-verbal Ability | 0.085 (1.008) | -0.099 (0.981) | <0.001 |
| Mathematics | 0.069 (0.952) | -0.081 (1.048) | <0.001 |

Supplementary Table 2. The results of curvilinear regression analysis for the association between *COMT* diplotype and cognitive function at ages 8 years and 15 years in boys and girls

| **Cognitive measures** | COMT diplotype | Boys | | | Girls | | |
| --- | --- | --- | --- | --- | --- | --- | --- |
|  |  | N | β | *P* | N | β | *P* |
| **Age 8** |  |  |  |  |  |  |  |
| Word reading | linear | 1012 | -0.176 | 0.29 | 1033 | 0.010 | 0.95 |
|  | quadratic |  | 0.031 | 0.35 |  | -0.004 | 0.88 |
| Vocabulary | linear | 1012 | -0.058 | 0.72 | 1033 | 0.177 | 0.27 |
|  | quadratic |  | 0.008 | 0.80 |  | -0.039 | 0.22 |
| Reading comprehension | linear | 1012 | -0.174 | 0.30 | 1033 | 0.173 | 0.26 |
|  | quadratic |  | 0.031 | 0.33 |  | -0.035 | 0.25 |
| Picture intelligence | linear | 1017 | 0.050 | 0.74 | 1033 | 0.057 | 0.72 |
|  | quadratic |  | -0.007 | 0.81 |  | -0.013 | 0.68 |
| **Age 15** |  |  |  |  |  |  |  |
| Reading Comprehension | linear | 1015 | -0.235 | 0.15 |  | -0.043 | 0.79 |
|  | quadratic |  | 0.034 | 0.21 | 1034 | 0.002 | 0.93 |
| AH4 Verbal Ability | linear | 1017 | -0.218 | 0.18 |  | 0.050 | 0.75 |
|  | quadratic |  | 0.035 | 0.28 | 1034 | -0.008 | 0.80 |
| AH4 Non-verbal Ability | linear | 1016 | 0.121 | 0.45 |  | -0.122 | 0.44 |
|  | quadratic |  | -0.026 | 0.42 | 1035 | 0.017 | 0.59 |
| Mathematics | linear | 1016 | -0.216 | 0.20 |  | -0.139 | 0.34 |
|  | quadratic |  | 0.040 | 0.23 | 1034 | 0.023 | 0.42 |
|  |  |  |  |  |  |  |  |
